# Allergic Fungal Rhinosinusitis in a 12-Year-Old Male Resulting in the Remodeling of Cribriform Plate With Protrusion Into the Anterior Cranial Fossa: A Case Report

**DOI:** 10.7759/cureus.49561

**Published:** 2023-11-28

**Authors:** Nicholas M McGhee, Savannah Groves, Carlton Homan, Christine Franzese

**Affiliations:** 1 Otolaryngology, University of Missouri School of Medicine, Columbia, USA

**Keywords:** invasive fungal infections, allergic sinusitis, proptosis, pediatric, allergic fungal rhinosinusitis

## Abstract

Allergic fungal rhinosinusitis (AFRS) in the pediatric population is a rare pathologic entity, and it is characterized by a type I hypersensitivity reaction to sinus fungi promoting the development of eosinophilic inflammation and thickened mucin. In the United States, a higher prevalence of AFRS is found among younger populations, African Americans, in counties characterized by elevated poverty rates, and patients without insurance or those reliant on Medicaid. Early clinical suspicion is essential for the timely diagnosis of this condition and to prevent the dissemination of the disease, thereby achieving a favorable prognosis. We report a case of a 12-year-old African American male who presented with the gradual onset of asymptomatic proptosis and seasonal allergy symptoms resulting in unilateral relative afferent pupillary defect and was ultimately diagnosed with AFRS.

## Introduction

Allergic fungal rhinosinusitis (AFRS), a subtype of chronic rhinosinusitis (CRS), is characterized by a type I hypersensitivity reaction to sinus fungi promoting the development of eosinophilic inflammation and thickened mucin [1]. AFRS often manifests clinically as nasal discharge, nasal obstruction, nasal polyps, anosmia, and headaches. However, in pediatric patients, AFRS often presents with facial and ocular symptoms such as sinus pain and proptosis [2]. CRS in the pediatric population is associated with a multitude of diverse etiologies, which help guide the course of treatment. In this report, we discuss an uncommon case of a 12-year-old male presenting with proptosis and a left relative afferent pupillary defect who was eventually determined to have chronic sinus disease consistent with AFRS, a rare clinical entity that requires a high index of suspicion and early recognition so that the challenging treatment often involving both medical and surgical intervention can be promptly initiated.

## Case presentation

**Figure 1:**
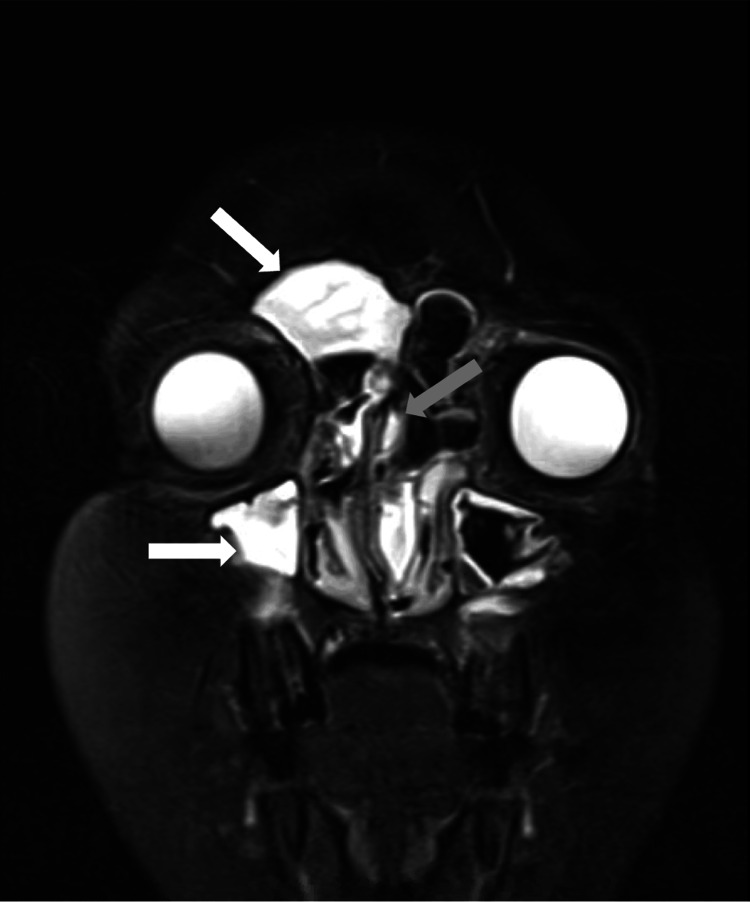
Coronal T2-weighted MRI of face and neck The image demonstrates diffuse hyperintense signals within the paranasal sinuses. There is opacification of the right frontal sinus and right maxillary sinus (white arrows). Expansion of the right and left ethmoid sinuses is demonstrated as well (grey arrow) MRI: magnetic resonance imaging

A 12-year-old African American male presented to the emergency department for advanced imaging after the evaluation of a left relative afferent pupillary defect discovered in an ophthalmology clinic the same day. The patient’s parents had noticed left eye proptosis about four weeks prior with the onset of seasonal allergy symptoms, of which the patient had a lifetime history. The patient's past medical history was also significant for acanthosis nigricans, hidradenitis suppurativa, elevated BMI (99th percentile for age), and an episode of preseptal cellulitis 10 years prior. Of note, the patient and his family had recently relocated from the Northeast region of the United States to the Midwest. At presentation, the patient was asymptomatic and denied any ocular pain, vision or hearing changes, ocular or nasal drainage, anosmia, and fever. An MRI without contrast of the face, neck, and orbit was ordered and results demonstrated diffuse T2 hyperintensities and multiple hypointensities in the frontal sinuses, ethmoid air cells, and maxillary sinuses (Figure 1).

**Figure 2:**
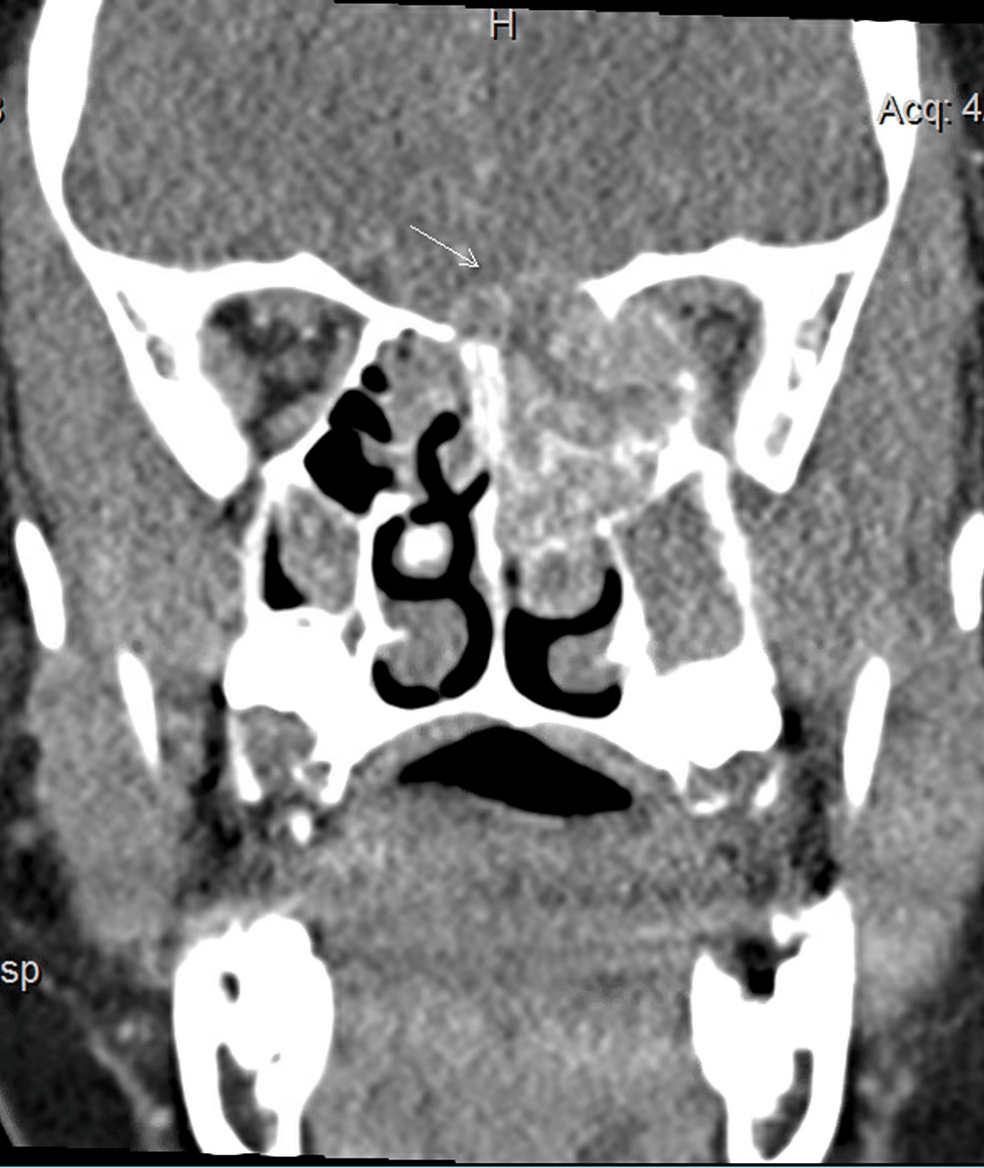
Maxillofacial CT scan of face and neck with contrast The image demonstrates significant diffuse sinus opacification (left greater than right) with areas of osseous dehiscence along the left medial orbital wall and extension into the left upper nasal space. Superiorly, there is erosion of the floor of the anterior cranial fossa with extension of the ethmoid air cell opacification into the left and right frontal lobes (arrow) CT: computed tomography

Imaging in the emergency department was consistent with a presumptive diagnosis of allergic fungal sinusitis with bony remodeling of the cribriform plate and protrusion into the anterior cranial fossa. An additional maxillofacial CT with contrast was consistent with these findings, demonstrating significant diffuse sinus opacification (left greater than right) with areas of osseous dehiscence along the left medial orbital wall and extension into the left upper nasal space (Figure 2).

**Figure 3:**
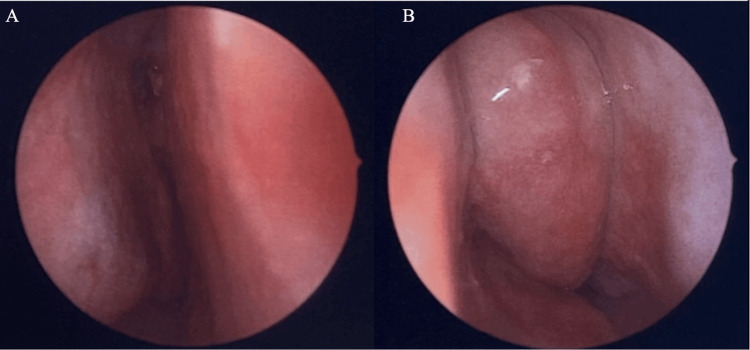
Endoscopic images of the left nasal cavity, with the close-up featuring a concha bullosa (A), with polypoid changes (B)

The patient was subsequently taken to the operating room under the care of Otolaryngology, and a flexible laryngoscopy was performed. He was found to have a chronic and expansile process of left-sided sinuses with mass effect on the lamina papyracea and the skull base at the fovea ethmoidalis (Figure 3).

The diagnosis of diffuse AFRS with bony expansion and dehiscence was made, and oral steroids and subsequent surgery were recommended based on this diagnosis. The patient was considered high-risk for surgery given the expansile nature of his disease and osseous involvement with an increased risk of injury to his left eye and brain. 

**Figure 4:**
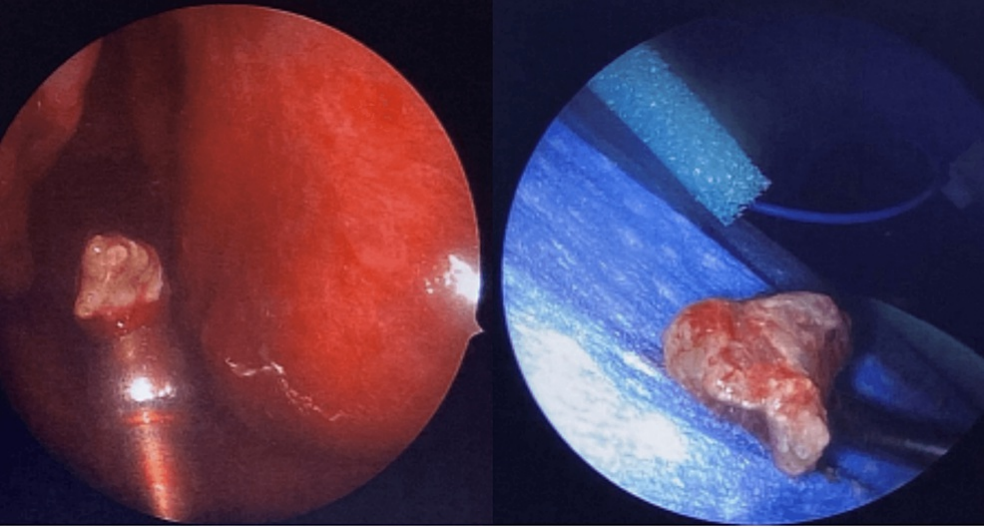
Fungal mucin extracted from the patient’s sinus cavities

The patient was treated surgically with bilateral maxillary antrostomy, bilateral total ethmoidectomy, bilateral frontal sinusotomy, bilateral concha bullosa resection, bilateral inferior turbinate outfracture, and left sphenoidotomy with the removal of fungal contents and subsequent irrigation (Figure 4).

Pathology specimens collected intraoperatively revealed changes consistent with allergic fungal sinusitis. No cultures were obtained from surgical pathology specimens. At one-week and one-month postoperative clinic visits, nasal endoscopies revealed grade 1 polyps of the left middle meatus with swelling of the frontal outflow tract. To date, the patient denies any residual vision or hearing deficits and also denies any other complications in the postoperative period. Final recommendations were made for the regular use of fluticasone nasal spray and nasal saline irrigation and for consideration of starting oral steroid regimens in the future.

## Discussion

AFRS is a hypersensitivity reaction to the presence of extra-mucosal fungal epitopes in the sinuses, resulting in eosinophilic inflammation, thickened mucin, and nasal polyps. The disease process in the pediatric population is complicated by anatomic sinuses continuing to develop well into adolescence; the frontal and sphenoid sinuses do not reach structural maturity approximately until the ages of 14 and 19 years, respectively [1]. Children tend to have smaller sinus ostia and enlarged adenoids, creating challenges in mucociliary clearance and predisposing this population to obstruction and sinus disease [1]. The younger population also tends to have a higher incidence of predisposing triggers for this disease, including an increased number of viral infections, allergic rhinitis, primary immunodeficiency syndromes, and ciliary dysmotility disorders [1]. All of these factors in combination tend to make pediatric CRS and AFRS both more challenging to diagnose and manage in the long term. 

AFRS most commonly presents with nasal obstruction, nasal discharge and rhinorrhea, anosmia, and headaches. In the pediatric population, a much larger proportion of patients tend to present with facial skeletal abnormalities, osseous involvement, and ocular manifestations, such as proptosis [2]. One study found that 25% of pediatric patients diagnosed with AFRS presented with proptosis, while another found that closer to 50% of pediatric patients diagnosed with AFRS presented with such symptoms when compared to adult patients with the same diagnosis [2,3].

The clinical workup of sinus disease in the pediatric population focuses initially on establishing the diagnosis and then determining the etiology of the disease. The initial workup includes laboratory evaluation, commonly revealing elevated IgE and eosinophilia, and radiographic imaging of sinusitis [3]. Due to higher rates of atopy in children with refractory sinus disease, allergy testing for aeroallergens, both perennial and seasonal, early in the course of investigations is often recommended [1]. Advanced imaging is employed in cases with evidence of a sinus mass or any intracranial or orbital complications [1]. Nasal endoscopy is almost always used in the diagnostic workup, demonstrating posterior pharyngeal drainage, edema, and discharge - endoscopy may also be helpful in uncovering the etiology by discovering structural abnormalities, such as adenoid enlargement, nasal polyps, and septal deviation [1]. The most widely used diagnostic criteria are that of Bent and Kuhn, which are as follows: (1) type I hypersensitivity to fungi, (2) nasal polyposis, (3) characteristic radiograph findings, (4) eosinophilic mucin, and (5) positive fungal stain or culture of sinus contents [4]. While the presence of fungi in allergic mucin is crucial for the diagnosis of AFRS, this is often challenging since hyphae tend to be sparse within the sinus contents [5]. This makes disease categorization difficult, especially given the significant overlap that exists between AFRS and eosinophilic mucin rhinosinusitis (EMRS), another subtype of CRS. 

Initial management is typically pharmacologic and aims to minimize inflammation, improve sinus drainage, and eradicate any pathogens. The first-line therapy usually involves empiric oral antibiotics against routine pathogens, such as S. pneumoniae, H. influenzae, and M. catarrhalis, in conjunction with oral steroids. Nasal saline irrigation is recommended for all AFRS patients, as well as allergen immunotherapy depending on the results of allergy testing [1]. Endoscopic sinus surgery is performed in a large segment of CRS patients, and AFRS patients specifically tend to require multiple surgeries [3]. Surgical intervention involves the removal of any nasal polyps, fungal debris, and inflamed tissue products [1]. The gold standard of surgical intervention includes postoperative regimens of nasal irrigations, postoperative endoscopic cleanings, and systemic corticosteroids [4]. Recommendations specific to the pediatric population include aggressive weaning of postoperative systemic steroid therapy to minimize complications, especially potential negative long-term growth effects [4].

The primary prognostic issue in both adult and pediatric populations is the high rate of disease recurrence. Some studies found pediatric disease relapse rates of around 55% at the one-year follow-up in patients who received endoscopic sinus surgery followed by nasal irrigation, endoscopic cleanings, and systemic steroid therapy [3]. Without postoperative adjuvant therapy, recurrence rates can be as high as 100% [2].

## Conclusions

AFRS in the pediatric population is a rare clinical entity. Early recognition and diagnosis of AFRS are essential to prevent the systemic progression of the disease. Prompt medical and surgical intervention should be carried out, including the initiation of systemic antifungal therapy, surgical extraction of fungal and mucin aggregates, and continued postoperative treatments including saline rinses and fluticasone nasal spray. A delay in diagnosis can lead to disseminated disease, which carries a worse prognosis. Our case report described a pediatric patient with chronic AFRS managed successfully with surgical intervention as well as postoperative debridements and at-home therapies. Clinicians should be aware that anti-fungal and anti-allergy therapies alone are often not sufficient for the resolution of infection, and successful treatment relies on surgical intervention, postoperative endoscopies, and at-home pharmacologic treatments as well. Clinicians should also maintain a high index of suspicion for the dissemination of the infection and utilize appropriate laboratory and imaging studies to monitor this early in the course of patient care.
